# Mechanisms from Growth Mindset to Psychological Well-Being of Chinese Primary School Students: The Serial Mediating Role of Grit and Academic Self-Efficacy

**DOI:** 10.3390/bs15050621

**Published:** 2025-05-02

**Authors:** Yicen Meng, Yan Sun, Lizhu Yang, Yasmin Othman Mydin

**Affiliations:** 1 School of Psychology, Liaoning Normal University, Dalian 116029, China; 2 School of Educational Studies, Universiti Sains Malaysia, George Town 11800, Penang, Malaysia

**Keywords:** growth mindset, psychological well-being, grit, academic self-efficacy, serial mediation, primary school students

## Abstract

The psychological well-being of primary school students holds significant value for their academic success and overall life development. A growth mindset is one of the critical factors influencing psychological well-being, yet the mechanisms through which a growth mindset exerts its impact remain underexplored. This study investigates the relationship between growth mindset and psychological well-being, focusing on the mediating role of grit and academic self-efficacy among primary school students. A sample of 301 students from a primary school in Northeast China was selected via convenience sampling. Data were collected using validated questionnaires and analyzed using SPSS 26.0. Serial mediation analysis was performed with Hayes’ PROCESS macro (Model 6, V4.0). Results indicated that growth mindset indirectly predicted psychological well-being through grit and academic self-efficacy. Both constructs served as individual mediators and acted as serial mediators in the pathway from growth mindset to psychological well-being, fully bridging the connection. These findings suggest that fostering a growth mindset, along with grit and academic self-efficacy, can enhance psychological well-being among primary school students. Educators and policymakers are encouraged to implement interventions targeting these factors to support students’ overall development.

## 1. Introduction

The primary school stage is crucial for developing academic, social, and emotional competencies (Nagaoka et al., 2015). Psychological well-being, which is thought to represent optimal human functioning (Diener, 2009), has a vital role in shaping students’ social interactions, self-esteem, and long-term development (Diener et al., 2006; Gomez-Lopez et al., 2022; Singhal & Prakash, 2021). A strong sense of psychological well-being enables children to manage stress, develop positive relationships with peers, and build confidence for future academic and life challenges (Y. H. Kim & Koh, 2014; Klainin-Yobas et al., 2021; Sabouripour et al., 2021). However, in China, the persistent pressures of a focus on exams and a dominant culture of success-oriented values have significantly jeopardized the well-being of primary school students (Lu et al., 2021; Cheng & Lin, 2023). With frequent exams and rising academic expectations, young children face unprecedented levels of pressures, which result in a lack of time for socializing with peers, leading to emotional disturbances such as anxiety, stress, and loneliness (Du et al., 2023; Li & Wang, 2024; Luo, 2018; J. Xu et al., 2023). In light of these potential detrimental outcomes, it becomes imperative to investigate the factors and mechanisms that can enhance the psychological well-being of primary school students (Tejada-Gallardo et al., 2020), particularly within the framework of the Chinese educational system.

Among the myriad of factors influencing psychological well-being, growth mindset —the conviction that abilities and intelligence are malleable and can be cultivated through effort, learning, and perseverance rather than being immutable traits (Dweck, 2006)—has garnered increasing scholarly attention (M. S. Kim & Park, 2021; Savvides & Bond, 2021; Zeng et al., 2016). Research underscores that fostering a growth mindset bolsters resilience, mitigates anxiety, and enhances well-being by encouraging children to perceive challenges as opportunities for development rather than as threats (Dweck, 2006; Yeager & Dweck, 2012; Zeng et al., 2016). However, existing research on growth mindset has been predominantly concerned with adolescents and young adults in high schools and universities, with limited attention given to primary school-aged children (King & Trinidad, 2021), especially in the Chinese culture context. In parallel, earlier studies on growth mindset have primarily examined its relationship with outcome variables such as learning performance, academic achievement, and motivation (Macnamara & Burgoyne, 2023; Sun et al., 2021), while neglecting to explore its predicting role in psychological well-being as a belief-driven instinct motivation and the underlying mechanisms of its influence. Furthermore, research suggests that grit—the perseverance and passion for long-term goals (A. L. Duckworth et al., 2007)—and academic self-efficacy—the belief towards one’s abilities to succeed academically (Schunk, 1989)—might function as mediators between growth mindset and psychological well-being (H. Zhao et al., 2023; W. Zhao et al., 2024). Thus, this study investigates the serial mediation effects of grit and academic self-efficacy on the relationship between growth mindset and psychological well-being. By focusing on primary school students, it addresses a critical research gap and sheds light on mechanisms that enhance both academic and emotional development, providing actionable insights to support students in navigating increasing challenges.

## 2. Literature Review and Hypotheses

### 2.1. Growth Mindset and Psychological Well-Being

Growth mindset, or Implicit Theories, refers to the belief that abilities and intelligence are malleable and can be developed through effort (Dweck & Leggett, 1988; Dweck et al., 1995; Molden & Dweck, 2006; Yeager & Dweck, 2012). This contrasts with a fixed mindset, where individuals believe that their abilities are static and unchangeable (Dweck, 2006). While growth mindset has been extensively studied in relation to academic achievement and motivation (Yeager et al., 2019; Macnamara & Burgoyne, 2023; J. Zhang et al., 2024; W. Zhao et al., 2024), recent research highlights its significant role in fostering psychological well-being across diverse populations (Zeng et al., 2016; Macnamara & Burgoyne, 2023). Psychological well-being encompasses multiple dimensions, such as meaning and purpose (Ryff, 1989; Ryff & Keyes, 1995; Seligman, 2011), supportive and rewarding relationships (Ryff, 1989; Ryff & Keyes, 1995; Deci & Ryan, 2000), competency (Deci & Ryan, 2000; Ryff, 1989; Ryff & Keyes, 1995), self-acceptance (Maslow, 1954; Ryff, 1989; Ryff & Keyes, 1995), being respected (Maslow, 1954; Ryff, 1989; Ryff & Keyes, 1995), etc., and is closely tied to students’ mindset. While fixed mindset students avoid challenges to protect self-esteem, growth mindset students embrace challenges as opportunities for improvement (Dweck & Leggett, 1988). These divergent reactions naturally result in differences in happiness and general well-being and are a good illustration of the role of the mindset in determining students’ well-being.

Recent studies have indeed confirmed that growth mindset relates positively to psychological well-being (Himmelberger et al., 2024; Zeng et al., 2016). For instance, Whittington et al. (2017) found that veterinary students with a fixed mindset scored significantly lower compared to those with a growth mindset on the scale of psychological well-being. In addition, Mosanya (2021) identifies growth mindset as a buffer in alleviating the effect of loneliness and academic stress caused by social isolation, thereby fostering resilience. Supporting this, Chan et al.’s (2022) investigation into domain-specific growth mindsets among adolescents in Hong Kong reveals that growth mindsets, particularly in ability and relationship domains, significantly influence psychological well-being. These findings collectively reveal the pervasive impact of growth mindset on psychological well-being across diverse student populations and emphasize its relevance in educational contexts.

However, the connection between mindset and psychological well-being is not always straightforward, and it has occasionally been shown to be indirect (Zeng et al., 2016). H. Zhao et al. (2023) posited that growth mindset influences adolescents’ well-being with the mediators of self-efficacy and gratitude. In a recent study in China, Y. Xu and Wang (2024) found that a growth mindset enhances psychological well-being by elevating enthusiasm. This line of research illuminates how growth mindset affects students’ psychological well-being through non-cognitive and motivational constructs. Hence, we propose the following:

**Hypothesis** **1.** *Growth mindset positively predicts psychological well-being.*

### 2.2. Grit as a Mediator Between Growth Mindset and Psychological Well-Being

Grit, which has gained considerable attention recently, may play a significant role as a non-cognitive personality-related construct in connecting growth mindset and psychological well-being. A. L. Duckworth et al. (2007) defined grit as persistent effort and persistent enthusiasm towards long-term goals, even when confronted with adversity and difficulties. This personality trait allows individuals to pursue their long-term goals with consistent interest and enthusiasm over an extended period. Students with a growth mindset focus on self-improvement and personal development, actively seek new opportunities, persist in their efforts, and embrace challenges, showcasing exceptional perseverance and a remarkable level of grit in pursuing long-term goals (Blackwell et al., 2007; Dweck, 2006; A. Duckworth, 2016).

The impact of a growth mindset on grit has been substantiated by an expanding body of research. For instance, Y. Zhao et al. (2018) identified that a growth mindset affects grit through the mediating role of learning motivation. Consistent with this, Y. Liu et al. (2021) demonstrated that grit partially mediates the relationship between a growth mindset and subjective well-being among Chinese adolescents, emphasizing that grit serves as a critical conduit through which a growth mindset enhances students’ well-being. In a recent longitudinal study involving Chinese adolescents, T. Zhang et al. (2022) reported that growth mindset and grit exhibit a reciprocal relationship, with each trait fostering the other’s development—a pattern echoing the findings of Park et al. (2020) among U.S. adolescents. Additionally, Y. Zhao et al. (2022) established that a growth mindset correlates positively with all dimensions of grit, further indicating that it bolsters grit by shaping individuals’ future-oriented perspectives and achievement motivation. These findings elucidate observations that students who perceive their school culture as promoting a mastery-oriented approach to learning and skill development tend to display heightened levels of grit (Park et al., 2018). Moreover, cognitive neuroscience evidence offers groundbreaking insights into the neuroanatomical underpinnings of grit, revealing through structural magnetic resonance imaging that a growth mindset is instrumental in cultivating higher levels of grit (S. Wang et al., 2018). In summary, students’ growth mindset emerges as a pivotal predictor of grit.

Recent research emphasizes the pivotal role of grit in fostering psychological well-being. For instance, Shah and Deshpande (2022) identified a robust positive correlation between grit and psychological well-being among college students in India. Similarly, L. Tang and Zhu (2024) demonstrated that grit functions as a significant predictor of psychological well-being among EFL students in China. Furthermore, Andales et al. (2025) found that grit was a significant predictor of psychological well-being among college students. Notably, individuals exhibiting higher levels of grit tend to display enhanced resilience, experience elevated positive emotions, and attain superior psychological well-being, as such traits enable them to persevere through challenging circumstances (A. Duckworth, 2016). Moreover, recent findings revealed that grit acts as a mediator in the association between a growth mindset and occupational well-being among Chinese EFL teachers (J. He et al., 2023). Collectively, these empirical findings highlight grit as an indispensable determinant of long-term well-being, fostering perseverance in adversity and sustained commitment to personal and professional goals over time.

From the reviewed literature, it is clear that both growth mindset and grit are substantially important contributors to predicting psychological well-being. However, despite these findings, little work has been performed to examine the underlying mechanisms, especially among younger age groups like Chinese primary school students. Therefore, the following research hypothesis is put forward:

**Hypothesis** **2.** *Grit mediates the relationship between growth mindset and psychological well-being.*

### 2.3. Academic Self-Efficacy as a Mediator Between Growth Mindset and Psychological Well-Being

Previous studies have already established that growth mindset can lead to better effects on academic performance and life satisfaction through academic self-efficacy among primary school students, which again highlights the positive link of the growth mindset with academic self-efficacy (Diao et al., 2020). Academic self-efficacy refers to students’ beliefs regarding their abilities to complete specific learning tasks successfully (Schunk, 1989), and it has proved to play a crucial role in shaping students’ engagement and motivation (Meng & Zhang, 2023; Schunk, 1991). According to Social Cognitive Theory, self-efficacy impacts how individuals approach tasks, set goals, and persist when confronted with challenges. It is shaped by a variety of sources, including performance attainments, enactive attainments, verbal persuasion, and physiological states (Bandura, 1977). Individuals with a growth mindset regard their abilities and intelligence as something that can be improved through continuous effort and learning, which strengthens their sense of control by actively working towards their mastery goals (Dweck & Leggett, 1988). This authentic mastery experience is one of the most influential sources of efficacy (Bandura, 1977). On this basis, those with a growth mindset are more likely to engage proactively with challenges, believing that their efforts will lead to improvement. According to the Stress and Coping Theory (Lazarus & Folkman, 1984), individuals’ primary and secondary appraisals of academic challenges shape their coping responses; growth mindset and academic self-efficacy enhance secondary appraisal by strengthening students’ confidence in their coping resources, leading to more frequent use of problem-focused coping strategies that foster a deeper sense of meaning in their learning experiences. For instance, McWilliams (2014) surveyed grade nine students and discovered that those with a growth mindset exhibited a clearer understanding of their academic abilities and had stronger academic self-efficacy. However, while growth mindset fosters positive academic outcomes, the detrimental effects of the fixed mindset cannot be overlooked. Jiang et al. (2019) demonstrated that fixed mindset, which focuses on innate abilities, reduced academic self-efficacy and negatively affected students’ engagement in mathematics learning.

Empirical evidence from intervention studies also supports the effect of growth mindset on academic self-efficacy. For example, Meijerink et al. (2016) found that by altering students’ mindsets, it was possible to increase their self-efficacy in terms of their willingness to study STEM (science, technology, engineering, and math)-related projects, which could in turn influence the choice of research on these projects. Fleurizard and Young (2018) conducted a single-session growth mindset intervention with university students involved in mathematics tutoring, showing that even a 20-min intervention can lead to significant improvements in their mathematics exam scores and self-efficacy. Similarly, Burnette et al. (2019) found that growth mindset interventions in undergraduates brought about higher self-efficacy and task persistence, particularly in key academic assignments, compared to controls. These findings provide preliminary evidence of the beneficial effect of growth mindset on academic self-efficacy, supporting Dweck’s (2002) assertion that “children’s beliefs about their abilities have the strongest direct influence on their performance”, thus reinforcing the relationship between mindset and self-efficacy.

Academic self-efficacy and psychological well-being have been widely studied for their interrelation. According to Lazarus and Folkman’s (1984) Stress and Coping Theory, individuals assess stressors through a two-stage cognitive appraisal process. In the primary appraisal, a situation is evaluated for its potential threat, while in the secondary appraisal, individuals assess their resources to cope with the stressor. Academic self-efficacy plays a crucial role in this process, as students with high self-efficacy are more likely to perceive academic challenges as manageable, thereby reducing perceived stress and enhancing psychological well-being. This perspective suggests that academic self-efficacy not only influences students’ motivation and engagement but also their stress appraisal and coping mechanisms, contributing to their overall psychological well-being. As a consequence, students with high academic self-efficacy demonstrate autonomous motivation and better engagement in learning (Azila-Gbettor et al., 2021), maintain a positive purpose in life (Cai & Lian, 2022), have positive relationships with peers and teachers (Llorca et al., 2017; Y. Wang et al., 2024), and achieve their goals by personal growth (Cai & Lian, 2022; Gülşen & Şahin, 2023), enhancing a sense of self-acceptance (Peng, 2022), which benefits their psychological well-being (Rohmah et al., 2024). For instance, Siddiqui (2015) found that self-efficacy plays a significant role in promoting psychological well-being among undergraduates. Similarly, L. Tang and Zhu (2024) found that academic self-efficacy serves as an important predictor of psychological well-being, directly influencing the overall mental health of Chinese EFL students.

In summary, as previously discussed, a growth mindset has been demonstrated to promote psychological well-being through its positive influence on academic self-efficacy. Moreover, substantial empirical evidence supports the interplay between growth mindset, academic self-efficacy, and psychological well-being. However, few studies have specifically examined the mediating role of academic self-efficacy between growth mindset and psychological well-being. Based on discussion above, we propose the following:

**Hypothesis** **3.** *Academic self-efficacy mediates the relationship between the growth mindset and psychological well-being.*

### 2.4. The Serial Mediation by Grit and Academic Self-Efficacy

Grounded in Eudaimonism (Aristotle, 350 BCE/1984), psychological well-being places more emphasis on being meaningful (Ryff, 1989). Unlike subjective well-being, which emphasizes emotional expression and life satisfaction (Diener, 1984, 2000), psychological well-being extends beyond emotions to encompass self-realization (Ryff, 1989). For students, self-actualization primarily stems from academic success, which requires strong beliefs in their academic abilities and persistent effort (A. L. Duckworth et al., 2007; Schunk, 1989). Grit and self-efficacy are regarded as the key non-cognitive factors affecting student academic performance (A. L. Duckworth et al., 2019; Zimmerman, 2000); however, they manifest to varying extents. Grit is a personality-related construct (A. L. Duckworth et al., 2007), whereas self-efficacy is a sense of belief assessing one’s abilities within an educational context (Schunk, 1989). Research has shown that grit can influence EFL learners’ online engagement through online learning self-efficacy (Derakhshan & Fathi, 2024). In studies involving broader populations (such as nurses and university students), it has also been confirmed that grit can influence well-being through self-efficacy (Benny, 2024; Si et al., 2022). Furthermore, W. Zhao et al. (2024) confirmed the serial mediation of grit and academic self-efficacy by investigating the effect of growth mindset on academic delay of gratification among Chinese adolescents. That is to say, grit and self-efficacy not only contribute independently to academic success but also interact in complex ways, mediating the effects of other psychological constructs on students’ motivation and behavior. Based on our description above, we post our hypothesis as follows.

**Hypothesis** **4.** *Growth mindset influences psychological well-being via the serial mediations of grit and academic self-efficacy.*

### 2.5. The Current Study

**Figure 1:**
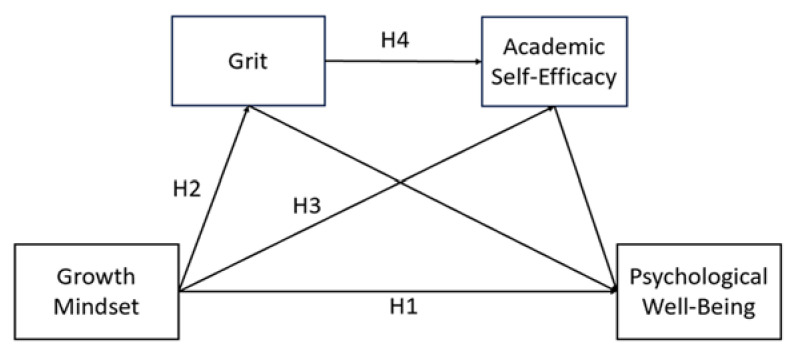
Research hypothesis model.

The present study, grounded in Implicit Theories and Stress and Coping Theory and with the support of previous research, aims to identify the mechanisms of grit and academic self-efficacy within a serial mediation model (see Figure 1) that illustrates how a growth mindset impacts the psychological well-being of primary school students in China. The current model aims to provide a new theoretical perspective in understanding the factors influencing psychological well-being and, based on empirical evidence, it proposes practical ways in which the well-being of primary school students could be improved. Growth mindset will enhance grit and academic self-efficacy, which subsequently enables students to deal with academic stress and challenges more positively, thus helping them improve their emotional regulation and sense of well-being.

As seen in Figure 1, the research hypothesis model demonstrates the serial mediation role of grit and academic self-efficacy in the relationship between growth mindset and psychological well-being.

## 3. Materials and Methods

### 3.1. Research Design and Approach

Quantitative methods, in the form of a questionnaire approach, are being adopted for the current study because its numerical and statistical findings are more able to be generalized (Creswell, 2015). The questionnaires will provide a very reliable means of assessing the relationship and identifying mediational effects through statistical analysis in use, which is commonly applied within growth mindset and academic development-related studies (Rammstedt et al., 2024). The scales applied in the present study had already been validated for their reliability and validity among Chinese students and were available in Chinese versions.

### 3.2. Participant and Procedures

This study utilized a questionnaire survey involving 320 fourth- and fifth-grade primary school students, chosen through convenience sampling from a specific region in Northeast China. Data collection took place in 2021. Convenience sampling was employed due to strict campus lockdown measures during the COVID-19 pandemic in 2021, which prevented external researchers from entering schools for data collection; consequently, participants were recruited from the school where the first author works to ensure timely and safe data collection. The participants’ ages ranged from 9 to 12 years (M = 9.79, SD = 0.70). Participants completed the survey within a 30-min class period, following detailed instructions provided by the researcher. However, 16 were excluded due to incomplete responses, straight-lining answers, and response times under one minute, and 3 outliers were detected using Mahalanobis Distance analysis, resulting in 301 valid responses being retained (effective rate = 94.0%). Table 1 displays the participants’ demographic statistics.

### 3.3. Materials

#### 3.3.1. Growth Mindset

The short version of Implicit Theories of Intelligence Scale for Children was used (Dweck, 1999). It is a self-evaluate scale for children age 9 and above. Four items (e.g., ‘You have a certain amount of intelligence and you can’t really do much to change it’) were assessed from strongly agree (1) to strongly disagree (6) on a Likert-style scale. A higher score reflects a stronger inclination toward a growth mindset, while a lower score suggests a fixed mindset. This scale has been applied to Chinese student populations, exhibiting adequate reliability and validity (Y. Zhao et al., 2018; Zeng et al., 2016; Niu et al., 2020). The coefficient Cronbach’s α is 0.722 in this study. Confirmatory factor analysis (CFA) (χ^2^/df = 0.361, CFI = 1.000, TLI = 1.018, RMSEA = 0.000) was within acceptable limits.

#### 3.3.2. Grit

The Chinese version of the Short Grit Scale (Grit-S) was applied to assess the level of grit (A. L. Duckworth & Quinn, 2009). This short scale comprises eight items with two dimensions: Perseverance of Effort (e.g., “I finish whatever I begin”) and Consistency of Interests (e.g., “I often set a goal but later choose to pursue a different one”). It has been translated into Chinese before, demonstrating strong reliability and validity among Chinese students (P. Yang, 2021). The scale items are responded to on a Likert-type scale ranging from 1 (“Not at all like me”) to 6 (“Very much like me”). In this study, the scale demonstrated good internal consistency, with a Cronbach’s alpha coefficient of 0.707. The CFA results (χ^2^/df = 1.401, CFI = 0.980, TLI = 0.970, RMSEA = 0.037) indicate an acceptable model fit.

#### 3.3.3. Academic Self-Efficacy

The Academic Self-Efficacy Subscale in the Motivated Strategies for Learning Questionnaire (MSLQ), developed by Pintrich and De Groot (1990) and revised by J. C. K. Lee et al. (2010) into a Chinese version, was used. The subscale consists seven items that describe the beliefs the students hold regarding their capacities on success academically, such as “I think I will do well compared with other students in my class”. Responses were rated using a Likert scale, with scores ranging from 1 (“Not at all true of me”) to 5 (“Very true of me”). The higher the score, the greater the students’ academic self-efficacy. The scale showed strong internal consistency in this study (Cronbach’s α = 0.873). The CFA results (χ^2^/df = 1.889, CFI = 0.988, TLI = 0.979, RMSEA = 0.054) indicate an acceptable model fit.

#### 3.3.4. Psychological Well-Being

The Psychological Well-Being Scale (PWB) was applied, which was also known as the Flourishing Scale (FS) (Diener et al., 2010). The Flourishing Scale is composed of eight items describing important aspects of human life, including positive relationships, a sense of competence, and a sense of meaning and purpose in life (e.g., “I lead a purposeful and meaningful life”). Each item of the FS is rated on a 1–7 Likert-type scale, ranging from Strong Disagreement to Strong Agreement. All items are worded positively. The Chinese version has exhibited satisfactory psychometric properties across a diverse sample of Chinese students (Duan & Xie, 2019; Lin, 2015; Tan et al., 2021). The reliability for this scale in this study was α = 0.825. The CFA results (χ^2^/df = 1.654, CFI = 0.980, TLI = 0.972, RMSEA = 0.047) indicate an acceptable model fit.

### 3.4. Data Analysis

Prior to data analysis, AMOS 26.0 was utilized for confirmatory factor analysis (CFA) of all research instruments employed in this study. SPSS 26.0 was used for data entry, data screening, and preliminary analysis. PROCESS macro plugin (Model 6) was used to test the serial mediation model (Hayes, 2012, 2018). This approach leverages an ordinary least squares (OLS) regression framework combined with a bootstrapping procedure, offering superior statistical power for mediation analyses compared to traditional three-step methods (Preacher & Hayes, 2008). This enabled an investigation of both the direct and indirect pathways through which a growth mindset impacts psychological well-being, mediated by grit and academic self-efficacy.

The analytical approach was structured into four phases: First, a set of preliminary analyses were implemented to ensure data integrity in alignment with established guidelines (Tabachnick & Fidell, 2019). These included managing missing values, detecting outliers through the Mahalanobis Distance method, conducting Harman’s single-factor test to evaluate common method bias, and performing normality checks using Skewness and Kurtosis indices. Multicollinearity was also assessed via the Variance Inflation Factor (VIF) and Tolerance values. Second, descriptive statistics were calculated to summarize key variables, encompassing means and standard deviations. Third, a Pearson correlation was performed to explore the relationships among growth mindset, psychological well-being, grit, and academic self-efficacy. Fourth, serial mediation analysis using Model 6 in the SPSS PROCESS macro assessed the mediating roles of grit and academic self-efficacy in the relationship between growth mindset and psychological well-being, with Bootstrap (5000 resamples) employed to validate the indirect effects.

## 4. Results

### 4.1. Common Method Bias Test

As all data in this study were collected via questionnaire measurement, there is a potential for common method bias. To assess this, an initial Harman’s single-factor test was conducted, revealing that four factors had eigenvalues above 1, with the highest variance accounted for by a single factor at 28.189%, which is below the commonly accepted threshold of 50%, indicating that common method bias is unlikely to be a concern in this study. This indicates that the variance is not primarily explained by a single factor, suggesting that common method bias (CMB) is unlikely to be a significant concern and thus supports the validity of the results (Podsakoff et al., 2003).

### 4.2. Normality and Multicollinearity Assumption Test

A normality test was conducted since many of the parametric statistical analyses require the data to be normally distributed (Ghasemi & Zahediasl, 2012; Tabachnick & Fidell, 2019). The normality test was performed by both visual inspection of histograms and analyzing skewness and kurtosis coefficients, which ranged between −2 and +2 for all research variables (See Table 3), confirming the normality assumption (Demir, 2022; Garren & Osborne, 2021). The multicollinearity check was performed to assess the degree of interrelationships among independent variables, as high multicollinearity could distort the results of accuracy in regression models by inflating standard errors and undermining statistical significance of predictors (Belsley et al., 1980; Field, 2013). Multicollinearity was analyzed using Variance Inflation Factor (VIF) and Tolerance metrics. The VIF values were less than 10 (ranging from 1.16 to 1.28), and the tolerance values exceeded 0.20 (ranging from 0.78 to 0.86). Therefore, it was determined that multicollinearity was not an issue (Martin & Bridgmon, 2012; Myers, 1990).

### 4.3. Validity and Reliability Testing

Reliability testing and Confirmatory Factor Analysis (CFA) were performed on the survey data to evaluate the reliability and validity of the measurement scales used in this research. The results are shown in Table 2.

### 4.4. Statistical Description and Correlation Analysis

Table 3 displays the results of the statistical and correlation analyses for the variables of growth mindset, grit, academic self-efficacy, and psychological well-being.

At the 0.01 significance level, growth mindset, academic self-efficacy, grit, and psychological well-being were significantly positively correlated. In contrast, gender and age showed no significant relationship with any of these variables.

### 4.5. Hypotheses and Serial Mediation Model Testing

To investigate whether grit and academic self-efficacy mediate the relationship between growth mindset and psychological well-being, Model 6 of the SPSS PROCESS macro plugins was used (Hayes, 2012). A bias-corrected non-parametric bootstrap method with 5000 resamples was used to assess the significance of the mediation impacts.

Table 4 provides the results. Regression analysis demonstrated that a growth mindset significantly positively predicted grit, β = 0.33, *p* < 0.001, and academic self-efficacy, β = 0.18, *p* < 0.01. In addition, grit positively predicted academic self-efficacy, β = 0.35, *p* < 0.001. After entering all variables into the regression model, the direct effect of a growth mindset on psychological well-being was found to be non-significant (β = 0.02, *p* > 0.05), suggesting a fully mediated relationship. Meanwhile, grit (β = 0.28, *p* < 0.001) and academic self-efficacy (β = 0.36, *p* < 0.001) were significant positive predictors of psychological well-being.

The results of mediation tests are summarized in Table 5. The mediation analysis outcomes demonstrated that grit and academic self-efficacy served as full mediators in the link between growth mindset and psychological well-being efficacy. Three indirect paths contributed to the mediating effect:

(1) Growth mindset → grit → psychological well-being; (2) growth mindset → academic self-efficacy → psychological well-being; (3) growth mindset → grit → academic self-efficacy → psychological well-being. The 95% confidence intervals for all three indirect paths, as can be seen in Table 5, do not include zero, confirming their significance.

**Figure 2:**
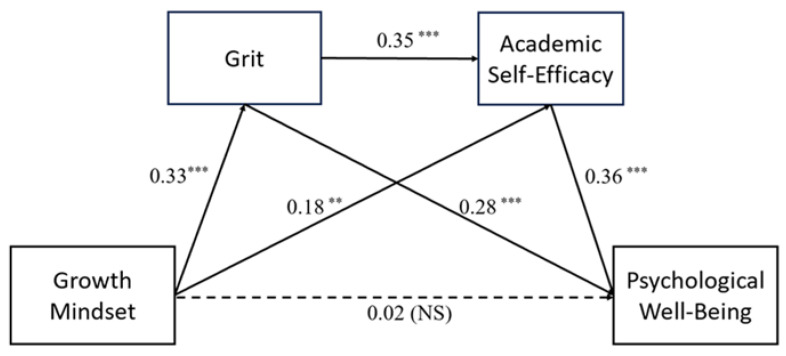
Mediation Effect Model. Notes: ^***^ *p* < 0.001, ^**^ *p* < 0.01. NS, not significant.

As seen in Figure 2, the mediation model suggested that the direct effect from growth mindset to psychological well-being was non-significant (β = 0.02, *p* > 0.05), which confirms a full mediation effect. These findings indicate that the relationship between growth mindset and psychological well-being was fully mediated by grit and academic self-efficacy. The model also confirms a serial mediation process: A growth mindset encourages grit, which then enhances academic self-efficacy, leading to improvements in psychological well-being. The findings point to the serial mediation structure of the model.

## 5. Discussion

This study, grounded in Implicit Theories and Stress and Coping Theory, utilized a serial mediation model to investigate the combined effect of a growth mindset, grit, and academic self-efficacy on the psychological well-being of primary school students. Specifically, it examined the mediations of grit and academic self-efficacy in linking a growth mindset to psychological well-being within the Chinese cultural context, thereby strengthening our understanding of the underlying mechanisms among these factors.

The results indicate that, in primary school, a growth mindset can significantly enhance students’ psychological well-being through three distinct mediation pathways: indirectly via the individual mediating effects of grit and academic self-efficacy, and indirectly through the serial mediation process in which a growth mindset increases grit, which subsequently enhances academic self-efficacy, ultimately leading to improved psychological well-being.

### 5.1. Correlations Between Growth Mindset and Psychological Well-Being

The results partially support Hypothesis 1, which proposed that growth mindset positively predicts psychological well-being. Specifically, the regression analysis (Table 4) revealed the direct path of growth mindset on psychological well-being was not statistically significant (β = 0.022, *p* > 0.05, 95% CI = [−0.071, 0.111]), indicating a growth mindset alone does not directly enhance psychological well-being in this sample. However, this finding does not necessarily undermine the importance of growth mindset in influencing psychological well-being. Consistent with prior research, growth mindset may exert its influence on well-being indirectly through other mechanisms or mediators (e.g., Zeng et al., 2016; Herdian et al., 2023). Indeed, as demonstrated by the mediation analyses (Table 5), growth mindset significantly predicts psychological well-being through grit and academic self-efficacy, as well as through the serial mediation of the two. These results suggest that the advantages of growth mindset for psychological well-being may not be immediate but operate through its capacity to promote perseverance—grit, and enhance students’ confidence in their academic abilities—academic self-efficacy.

Several explanations may account for the non-significant direct effect of growth mindset on psychological well-being. A plausible explanation lies in the nature of growth mindset as an internal cognitive belief that focuses on the malleability of abilities but has limited direct influence on psychological well-being, which is also shaped by external factors such as the learning environment (Lombas et al., 2019; Mustafa, 2020), parenting styles (Gul et al., 2021), socioeconomic status (Navarro-Carrillo et al., 2020), and family environment contexts (Bronfenbrenner, 1979; Shek, 1997), etc. As a belief system, a growth mindset primarily influences behaviors such as persistence and effort by adjusting self-regulation and coping strategies (Burnette et al., 2013; Hong et al., 1999). Individuals with a stronger growth mindset are more likely to persist through obstacles and maintain a positive attitude regarding their chances of future success. When encountering setbacks, they exhibit a more mastery-focused approach, seeking ways to enhance their abilities and performance, such as putting in additional effort or taking corrective actions. However, when external conditions are unsupportive, it becomes disconnected from the core psychological needs of autonomy, competence, and relatedness, which are negatively related with indicators of poor well-being (such as depression, apathy, etc.) (Ryan & Deci, 2000; M. Tang et al., 2020). For instance, growth-oriented students also put extra effort and persistence into improvement for better academic performance but may suffer from stress or frustration due to external pressures and resource constraints at the same time. This might underpin the lack of significant direct influences of growth mindset on psychological well-being reflected in our study.

Cultural and contextual factors may also affect the influence of a growth mindset on psychological well-being as well. In China’s highly competitive learning environments, students tend to prioritize external performance rewards rather than psychological benefits (Han et al., 2024; Xiang et al., 2019). Traditional educational models in China have long emphasized rote learning and academic excellence in schools (Cao, 2024), which may limit the opportunities to apply growth mindset principles beyond academic contexts and reduce its relevance to psychological well-being. To some extent, the fulfilment of autonomy, competence, and relatedness may be undermined in this context (Ryan & Deci, 2000). Furthermore, the structured, teacher-centered education system in China often restricts autonomy by restricting decision-making, focuses on standardized testing rather than personal progress, and provides fewer opportunities for collaborative learning, which may weaken relatedness and meaningful relationships (Chang et al., 2011; Luxia, 2007; X. Yang, 2013). These factors may further limit the extent to which a growth mindset can directly bolster students’ psychological well-being in such contexts.

Generally, these findings suggest that while growth mindset holds promise for improving students’ psychological well-being, its effects are largely mediated by contextual and individual factors such as grit and academic self-efficacy. The mediating roles are discussed as follows.

### 5.2. The Mediating Roles of Grit and Academic Self-Efficacy

The results clearly showed that grit and academic self-efficacy significantly mediated the relationship between growth mindset and psychological well-being among primary children. Specifically, both grit and academic self-efficacy operated as independent mediators, each making a distinct contribution to the pathway linking growth mindset to psychological well-being. Furthermore, these mediators also functioned in a sequential manner: a growth mindset enhanced grit, which in turn bolstered academic self-efficacy and ultimately fostering improved psychological well-being. This dual mediation highlights the interconnected complexity of these variables, aligning with prior research in educational psychology (A. L. Duckworth et al., 2007; Bandura, 1997). These findings corroborate research hypotheses 2, 3, and 4, establishing grit and academic self-efficacy (ASE) as critical mechanisms through which a growth mindset influences students’ psychological well-being.

Grit equips students with the perseverance necessary to navigate academic challenges, thereby cultivating competence and resilience that underpin psychological well-being (A. L. Duckworth et al., 2007; Yeager & Dweck, 2012). Similarly, ASE enhances students’ confidence in their ability to overcome obstacles and succeed in academic endeavors, reinforcing resilience and fostering positive experiences (Bandura, 1997; Diao et al., 2020).

Students with the growth mindset firmly believe that sustained effort leads to improvement, further reinforcing the critical role of grit and ASE in their psychological well-being. This mindset builds internal motivation and encourages sustained effort, a core element of grit (A. L. Duckworth et al., 2007; A. Duckworth & Gross, 2014). This sustained effort enhances their sense of control over the achievement of the goal. In the long term, they develop grit, which enables students to continue working toward their goals despite setbacks. This persistence leads to personal achievements and satisfaction (Yeager & Dweck, 2012; Zeng et al., 2016). Moreover, grit enhances student engagement in academic activities successfully since students can focus on their work and are thus less easily distracted. It would also contribute to psychological well-being with a sense of efficacy or mastery and resilience processes (M. Tang et al., 2020). Hence, grit is reinforced to be the mediator between the growth mindset and psychological well-being.

Similarly, academic self-efficacy (ASE) emerged as another important mediator. Academic self-efficacy has been widely recognized as a crucial factor in academic motivation and psychological well-being (Bandura, 1997; L. Tang & Zhu, 2024). Our study demonstrates that a growth mindset fosters academic self-efficacy (ASE) by reinforcing the belief that effort leads to success. Higher academic self-efficacy (ASE) equips students with the confidence to approach challenges, persist in their goals, and reduce anxiety, ultimately enhancing psychological well-being (X. He et al., 2023; Mohtasham et al., 2024; Siddiqui, 2015). According to Social Cognitive Theory (Bandura, 1977), mastery experiences—built through persistence—are central to developing self-efficacy. Growth mindset students engage actively with learning tasks, acquiring mastery experience, thus acquiring their sense of competence and control. As a result, this leads to an enhancement in psychological well-being. These findings are consistent with prior research. For example, studies have demonstrated that self-efficacy mediated the connection between growth mindset and adolescents’ sense of meaning in life, an essential dimension of psychological well-being (H. Zhao et al., 2023). Similarly, academic self-efficacy (ASE) promotes positive outcomes in learning and well-being (Ariani, 2021; X. He et al., 2023).

Both grit and ASE are proposed as important mechanisms in the pathways leading from a growth mindset to psychological well-being. This study extends existing studies by elaborating a more differentiated view on how cognitive beliefs affect psychological outcomes through motivational and behavioral mechanisms. However, these individual-level processes operate within broader contextual systems—such as school culture and parental support—that may further moderate or mediate these pathways. Future interventions may focus on fostering grit and academic self-efficacy (ASE) to promote student well-being in educational settings, while also considering the roles of school culture and parental support.

### 5.3. Contributions and Implications

This research explains how a growth mindset influences key factors like grit and academic self-efficacy. By exploring these connections, it shows how motivational factors work together to improve well-being. Moreover, the current research extends the literature by embedding grit and academic self-efficacy within a serial mediation model and showing how those two variables interact in linking growth mindset to psychological well-being. Although prior studies have separately examined the role of grit or self-efficacy in growth mindset and students’ well-being (Y. Liu et al., 2021; H. Zhao et al., 2023), the current research uniquely integrates these variables into a comprehensive chain mediation model. The findings support two powerful frameworks in educational psychology: Dweck’s Implicit Theories of Intelligence and Lazarus and Folkman’s (1984) Stress and Coping Theory.

Implicit Theories of Intelligence explains that individuals who believe their abilities can improve are more motivated to learn (Dweck, 2006). They see challenges as chances to grow. This mindset builds resilience and helps students cope with setbacks. Studies confirm this link. Students with growth mindsets show stronger resilience and psychological well-being (Yeager & Dweck, 2012; Burnette et al., 2022; Zeng et al., 2016). The current research also contributes to Stress and Coping Theory (Lazarus & Folkman, 1984). According to the Transactional Model of Stress and Coping, stress arises when students appraise academic demands as exceeding their coping resources. A growth mindset shifts primary appraisal by framing challenges as opportunities, and bolsters secondary appraisal by strengthening grit and academic self-efficacy, which in turn promote problem-focused coping (e.g., planning, sustained effort) and positive reappraisal of setbacks. These personal resources also encourage help-seeking and the building of social support networks, buffering the adverse effects of stress on psychological well-being (Cohen & Wills, 1985; Chemers et al., 2001). This integration shows how growth mindset can enhance well-being by improving both the appraisal of academic stressors and the effectiveness of coping strategies, yet future work should integrate ecological perspectives (Bronfenbrenner, 1979) to account for school- and family-level influences, both of which have been shown to significantly foster students’ growth mindsets (J. Yu et al., 2022; H. J. Lee & Mendoza, 2025), especially in cultures with higher collectivism.

Based on these aforementioned evidences, this study explores how these relationships develop in school settings. The results provide meaningful insights for educational practice and student development.

First, growth mindset affects students’ psychological well-being indirectly. Although its direct effect is non-significant, the effect works through the mediators of grit and academic self-efficacy. These mechanisms play an important role in enhancing the psychological well-being of primary school students. Educators should use strategies that promote goal-setting, persistence, and self-belief. For example, in Chinese primary schools—where the “Double Reduction” policy (General Office of the Central Committee of the Communist Party of China & General Office of the State Council, 2021) has shifted focus toward holistic development—teachers can implement collaborative projects that align with the national curriculum and local culture (e.g., community-based science fairs or group storytelling activities) to foster autonomy and relatedness while reducing exam-driven pressure. Guided reflection exercises and goal-oriented activities can likewise be embedded into reduced-homework schedules, leveraging after-school “quality education” programs now encouraged under the policy to provide dedicated time for implementing classroom-based socioemotional activities alongside other recommended strategies.

Secondly, grit plays a key role in linking a growth mindset to psychological well-being. Indeed, students with higher growth mindset compared to their peers are likely to persevere and be resilient. Through this way, the traits will help them solve different academic and personal challenges that will surely enhance well-being. In the Chinese context, an eight-session growth mindset intervention combining reading assignments with effort-based praise has been shown to increase resilience and engagement among 10–12-year-olds (C. Yu et al., 2024), demonstrating that structured grit-building workshops can be successfully integrated into local school timetables. Educators can build on this by offering mentorship programs, team-based activities, and setback-management workshops during the expanded non-academic hours created by the “Double Reduction” reforms.

Thirdly, academic self-efficacy is another mediator that shows and explains how a growth mindset enhances well-being. The belief in their capability to succeed through putting enough effort increases students’ confidence and promotes better mental and emotional states. Chinese schools can reinforce this by training teachers and parents to provide feedback focused on effort and strategy rather than innate ability. Creating a protective classroom environment where mistakes are treated as learning opportunities aligns with recent national guidelines on mental health in schools and supports the basic need for competence.

Fourth, the serial mediation of grit and academic self-efficacy acts to enhance the link between growth mindset and psychological well-being. This again supports a holistic approach. Policymakers should recommend programs that link growth mindset training with grit and development of self-efficacy. For instance, resilience workshops could be co-located with physical-education modules—now being prioritized in Chinese schools to promote holistic health—so that persistence-building exercises are paired with two-hour daily activity blocks, reinforcing both physical and psychological well-being.

Last but not least, this study contributes to broader educational research. It adds to emerging evidence on the interactional roles of psychological factors in education. The clarification of the mechanisms by which a growth mindset works introduces practical strategies to enhance students’ well-being. Further studies should explore the longitudinal impact brought about by the intervention of growth mindset, grit, and self-efficacy programs within the current policy framework. Across various education settings and levels of students, research may provide clear views on how reduced academic burden and enhanced quality-education programs jointly should be tailored to fit specific needs.

This study underlines that, in the context of Chinese culture, a growth mindset indirectly impacts psychological well-being among primary school students and specifies grit and academic self-efficacy as the pathways toward improvement in well-being. Furthermore, it has shown how a growth mindset supports stress and coping theory by reframing challenges as opportunities (thereby shifting primary appraisal) and by strengthening grit and academic self-efficacy (thereby bolstering secondary appraisal). Scholars, policymakers, and educators can take collective responsibility to address this complex interplay of issues. In so doing, they allow students to build resilience, gain confidence, and succeed better emotionally and academically.

### 5.4. Limitations and Future Recommendations

This study provides significant insights into the relationships between growth mindset, grit, academic self-efficacy, and psychological well-being among primary school students. However, a few limitations should be acknowledged.

First, this study utilized a convenience sampling approach. Data collection took place in 2021 during strict COVID-19 campus lockdowns, necessitating convenience-based recruitment which may have impacted data quality and participant representativeness. Though representative of the target population partly, this method might have contained sampling errors. Future research should use random sampling methods such as stratified or cluster sampling, with larger sample sizes. This would enhance external validity and better represent the target population.

The second limitation is that this study used a cross-sectional design, which restricts the establishment of causal relationships and does not capture dynamic changes in psychological well-being. Future studies should consider either longitudinal designs or experimental research. These approaches would show how students’ psychological well-being changes over grade level and extended periods. They would also afford an added understanding of how those variables develop and interact.

Third, this study concentrated on selected mediating relationships, which may not represent the intricacy of the interactions among variables. Other factors that could potentially come into play include teacher support, socioeconomic influences, and relationships with peers. These other factors are to be looked into in future studies to widen the perspective on how academic self-efficacy, grit, and psychological well-being may be related in various educational contexts.

Another limitation is the use of self-report measures. These have a number of response biases associated with them, such as the social desirability effect. Multiple assessment methods should be adopted in further studies, like ratings from teachers and parents. This would enhance the reliability of findings.

Finally, while our findings underscore the mediating functions of grit and self-efficacy, we recognize that broader contextual determinants—such as school culture and parental support—may further modulate these dynamics. Constructive school climates, distinguished by nurturing teacher–student interactions and a collective dedication to student well-being, have been demonstrated to improve overall mental health outcomes among elementary school students in China (C. Wang et al., 2018). Likewise, parental involvement in home- and school-based educational activities enhances primary students’ sense of security and autonomous motivation, potentially amplifying the efficacy of growth mindset on well-being indicators (X. Liu et al., 2024). Subsequent research should integrate factors such as school climate and family involvement to more comprehensively delineate the ecosystem shaping student psychological health.

## 6. Conclusions

This study deepens knowledge on the interrelationship between growth mindset, grit, academic self-efficacy, as well as psychological well-being among primary school students in China. The findings highlight several key conclusions. First, growth mindset did not exhibit a significant direct effect on psychological well-being, suggesting that its influence operates through indirect pathways, thereby partially supporting Hypothesis 1 (H1). Second, grit was identified as a mediator in the relationship between growth mindset and psychological well-being, aligning with Hypothesis 2 (H2). Third, academic self-efficacy also mediated this relationship, offering support for Hypothesis 3 (H3). Finally, grit and academic self-efficacy functioned as serial mediators, collectively bridging the effect of growth mindset on psychological well-being, which supports Hypothesis 4 (H4).

Specifically, students with a strong growth mindset demonstrated higher levels of grit and academic self-efficacy, leading to improvements in their psychological well-being. This study emphasized the role of growth mindset in enhancing psychological well-being among primary school students, and highlighted the need to foster grit and academic self-efficacy, as they are vital factors in this relationship. Strategies such as setting attainable learning goals, encouraging resilience and progress through small steps, and providing consistent support can help cultivate these qualities, thereby enhancing students’ well-being and academic development.

## Figures and Tables

**Table 1:** Sample demographics.

	Category	Frequency	Percentage
Gender	Male	171	56.8
	Female	130	43.2
Age	9	108	35.9
	10	153	50.8
	11	36	12.0
	12	4	1.3
Total	-	301	100

**Table 2:** Validity and reliability of the scales.

	χ^2^	df	χ^2^/df	CFI	TLI	RMSEA	SRMR	α
Growth Mindset	0.722	2	0.361	1.000	1.018	0.000 [0.00–0.08]	0.010	0.722
Grit	25.211	18	1.401	0.980	0.970	0.037 [0.00–0.07]	0.043	0.707
Academic Self-efficacy	22.673	12	1.889	0.988	0.979	0.054 [0.02–0.09]	0.025	0.873
Psychological Well-being	33.073	20	1.654	0.980	0.972	0.047 [0.01–0.07]	0.033	0.825

**Table 3:** Summary of descriptive statistics and correlations between variables.

Variables	M	SD	Skewness	Kurtosis	1	2	3	4	5	6
1. Gender	1.432	0.496	0.276	−1.937	1					
2. Age	9.790	0.699	0.554	0.048	−0.244 ^**^	1				
3. GM	4.390	1.236	−0.641	−0.281	−0.026	0.025	1			
4. Grit	4.116	0.812	−0.073	−0.370	−0.030	−0.104	0.330 ^**^	1		
5. ASE	3.604	0.826	−0.234	−0.020	0.012	−0.108	0.289 ^**^	0.415 ^**^	1	
6. PWB	5.467	1.088	−0.695	0.147	0.005	0.037	0.222 ^**^	0.426 ^**^	0.475 ^**^	1

Notes: ^**^ *p* < 0.01; n = 301; For gender, 1 = male and 2 = female. Abbreviations: GM, growth mindset; ASE, academic self-efficacy; PWB, psychological well-being.

**Table 4:** Results of regression analysis for the Serial Mediation Pathway.

Variables	Predictors	R	R^2^	F	β	t	LLCI	ULCI
Grit	GM	0.352	0.124	14.001 ^***^	0.331	6.099 ^***^	0.148	0.288
ASE	GM	0.452	0.204	18.974 ^***^	0.176	3.194 ^**^	0.045	0.190
	Grit				0.350	6.316 ^***^	0.254	0.467
PWB	GM	0.549	0.302	25.490 ^***^	0.022	0.424	−0.071	0.111
	GRIT				0.281	5.076 ^***^	0.231	0.523
	ASE				0.364	6.665 ^***^	0.337	0.620

Notes: ^***^ *p* < 0.001; ^**^ *p* < 0.01. Abbreviations: GM, growth mindset; ASE, academic self-efficacy; PWB, psychological well-being; LLCI, Lower Level of Confidence Interval; ULCI, Upper Level of Confidence Interval.

**Table 5:** Bootstrap Mediation Effect tests (n = 301).

Path	Effect Value	Boot SE	95% CI	
			LLCI	ULCI
GM → Grit→ PWB	0.082	0.021	0.044	0.124
GM → ASE → PWB	0.056	0.022	0.018	0.104
GM → Grit→ ASE → PWB	0.037	0.011	0.020	0.061
Direct effect (GM → PWB)	0.020	0.046	-0.071	0.111
Indirect effect	0.175	0.033	0.113	0.243
Total effect	0.195	0.050	0.097	0.293

Abbreviations: GM, growth mindset; ASE, academic self-efficacy; PWB, psychological well-being; Boot SE, Bootstrap Standard Error; CI, Confidence Interval; LLCI, Lower Level of Confidence Interval; ULCI, Upper Level of Confidence Interval.

## Data Availability

The datasets supporting the conclusions of this study are available from the corresponding author on reasonable request.

## Abbreviations

The following abbreviations are used in this manuscript:

GM	Growth mindset
ASE	Academic self-efficacy
PWB	Psychological well-being
SDT	Self-Determination Theory
CFA	Confirmatory Factor Analysis
